# Importance of Dietetics

**Published:** 1886-05

**Authors:** 


### IMPORTANCE OF DIETETICS.

Dr. W. Roberts, consulting physician to the Manchester Royal Infirmary, England, in the recent address published in the British Medical Journal devotes the entire hour to the subject of feeding the sick. We publish a portion of the articles;

Perhaps, of all the many duties which fall to the province of the medical practioner, there is none so common as the duty of regulating the diet of his patients. Whatever the disease may be from which the patient is suffering, and whatever special means may be indicated for his relief, the regulation of the diet is sure, sooner or later, to crop up as an integral part of the management of the case. Dietetics, therefore, cover more ground than any other branch of the healing art—they are also, perhaps, the most ancient branch. Hippocrats traces back the very origin of medicine to dietetics. "For" he says, 'the art of medicine would not have been invented at first, nor would it have been made a subject of suggestion, if, when men are indisposed, the same food, and other articles of regimen which they eat and drink when in good health, were proper for them, and if no others were preferable to these." Notwithstanding this universal applicability and this high antiquity, it must I think, be allowed that dietetics, except in a few special cases, are somewhat neglected in these days. The often contradictory advice which is tendered to invalids in regard to their diet by the several medical men who they may consult, betrays the want of a guiding principle, and of a general consensus of opinion in the medical profession on the subject of feeding the sick. This is, perhaps, not to be wondered at when it is considered how little systematic study is devoted to dietetics and how fragmentary is the instruction on this subject which is given to the student of medicine. So far as I know, there is no systematic teaching of dietetics, even on the most limited scale accorded to the student at any of our medical schools. He is left to pick up his knowledge on this subject, as best he may, during the earlier year of his practice; and he often ends by taking his own digestive organs as his type, and prescribes for his patients according to the likings and dislikings of his own stomach. This is, I need hardly say, a very unsatisfactory proceeding; for there is, perhaps, no subject on which individual experience is so fallacious a guide as dietetics, and none in regard to which it is more important to draw our inductions from a wide basis of facts.

In the matter of special diet for the sick, Dr. Roberts suggests what food are the most important, and of these milk, meat preparations, raw eggs, and the various gruels. Milk administered in the crude form is liable to coagulate in solid masses in the stomach. Eggs beaten up raw are not as digestible, as cooked but on into the duodenum and are obsorbed by the intestines.

Dr. Roberts concludes the address as follows:

Gentlemen, in bringing my remarks to a close I should like again to press for a more systematic and a more comprehensive study of dietetics. The effects of diet are profound and far-reaching, and exceeding subtle. Some inkling of this is got from the history of gout. You all know how slowly and how insidiously the agony diathesis is developed under the influence of diet, and how it may affect the descendants unto the third and fourth generation. The immediate effects of diet are often not the most important. Behind these are remote sequences of vital concern to the family and the nation. And it is not solely in regard to feeding the sick that a scientific knowledge of dietetics is useful. There are public questions of great moment, affecting the food-habits of the people, the consideration of which ought not to be dominated exclusively by popular opinion. In legislating on such questions, it is of the last importance to proceed on correct lines ; for it is certain that any policy which ignores the instincts of mankind and the laws of nature is foredoomed to failure. I believe that a comprehensive study of these questions from the side of history, and of natural history, would throw unexpected light on the issues involved, and furnish data of great value for the guidance of the legislator and of the social reformer.

